# Behavioural data on instar crab movement at different thermal acclimation

**DOI:** 10.1016/j.dib.2019.01.026

**Published:** 2019-01-18

**Authors:** Mohamad N. Azra, Mhd. Ikhwanuddin, Ambok Bolong Abol-Munafi

**Affiliations:** Institute of Tropical Aquaculture, Universiti Malaysia Terengganu, Malaysia

**Keywords:** Recorded video, Aquaculture, Water temperature

## Abstract

This article investigated how crabs responded to different culture temperatures especially dislocation before molting using a combination of large recording files and computer software. In this novel approach of video recording portunid crab behavioral data, crab culture was recorded at five different acclimation temperatures of 20, 24, 28, 32 and 36 °C. Crabs were reared until the instar stage before being acclimatized for video recording. Large video files (MPEG-TS) were then analyzed using the latest version of Solomon Coder software developed by A. Peter and programmed with Embarcadero^®^ Delphi^®^ XE [1]. Recorded data was analyzed by calculating and marking movements of crabs using the time sequence tool. Additionally, a total movement was counted 30 min before crabs molted from instar stage 8 to instar stage 9. Part of the data is associated with the research article “Thermal tolerance and locomotor activity of blue swimmer crab *Portunus pelagicus* instar reared at different temperatures” (Azra et al., 2018) [2] and provided here as raw data of Supplementary materials.

## Specifications table

**Table:** 

Subject area	Biology
More specific subject area	Thermal Behavior
Type of data	Table and figure
How data was acquired	Video camera (Sony, Model HDR-CX500E) and Solomon Coder Software (Version Beta 17.03.22)
Data format	Raw and analyzed
Experimental factors	Crabs were cultured until they molted into the next stage at different acclimation temperatures
Experimental features	Determine the locomotors activity (dislocation before molting) of the crabs in captive conditions
Data source location	Crustacean Group, Institute of Tropical Aquaculture, Universiti Malaysia Terengganu, Kuala Nerus, Terengganu, Malaysia
Related research article [2], [3]	– Azra, M.N., Chen, J.C., Ikhwanuddin, M. and Abol-Munafi, A.B. 2018. Thermal tolerance and locomotor activity of blue swimmer crab Portunus pelagicus instar reared at different temperatures. J. Therm. Biol. 74: 234–240. – Abol-Munafi, A.B. and Azra, M.N. 2018. Climate change and the crab aquaculture industry: Problems and challenges. J. Sustain. Sci. Manag. 13(2): 1–3

## Value of the data

• The behavioral data provided here can be used to established conventional patterns with other results and for further thermoregulation research.
• Behavioral changes might be one of the potential indicators on how cultured animals adapt to fluctuating environments especially water temperature.
• The effects of acclimation temperature on behavioral activity using video recording and software analysis will be useful for other researchers investigating adaptive evolution and phenotypic plasticity in climate change scenario.

## 1 Data

Included in this article are the raw data, descriptive data (means) and linear regression on the effects of different acclimation temperature on the locomotor behavior of the blue swimming crab, *Portunus pelagicus.* The selected temperatures of 20, 24, 28 (control), 32 and 36 °C were based on the previous research [2]. In brief, crabs prefer lower temperatures during the stocking of aquaculture ponds.

## 2 Experimental design, materials and methods

### 2.1 Broodstock collection and instar sources

**Fig. 1:**
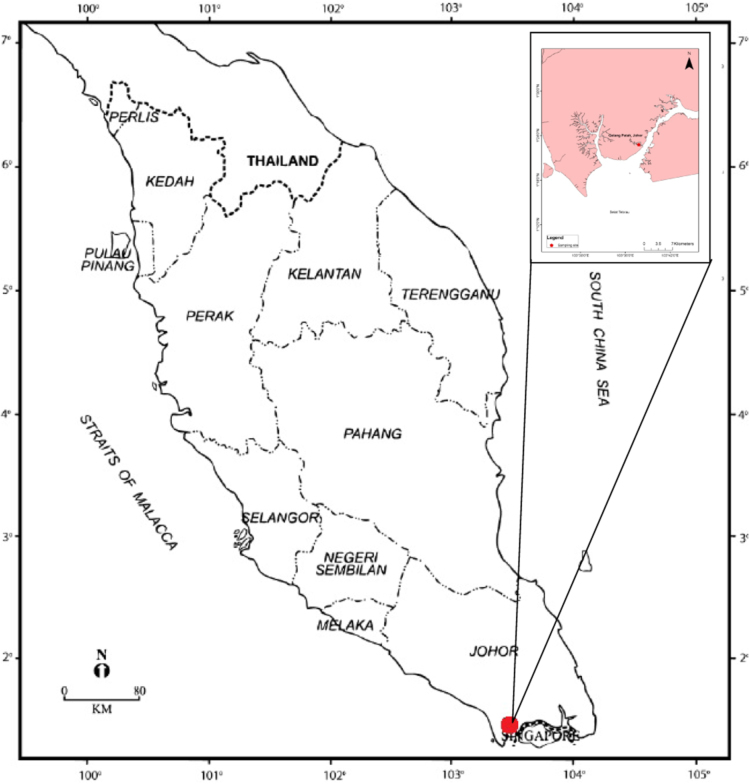
Map of the Peninsula Malaysia with location of sampling site, Pendas Jetty, Gelang Patah, Johor, and indicated as red dot in both maps.

Berried females were collected from the wild (Johor coastal water, Southern Peninsular Malaysia, Fig. 1) and transferred back to the hatchery at Institute of Tropical Aquaculture, Universiti Malaysia Terengganu and placed into hatching tanks (1000 L). Larval crabs were then collected and stocked in the larval culture tanks (100 L). Crabs were cultured until they reached megalopa stage [4], [5]. Crabs were then cultured individually (300–500 mL polystyrene cups) from instar stage 1 up until instar stage 8 for further analysis on behavioral changes at different water temperatures. Instar 8 was chosen because their size was suitable for further stocking in the pond [6].

### 2.2 Experimental setup, video recording and software application

**Fig. 2:**
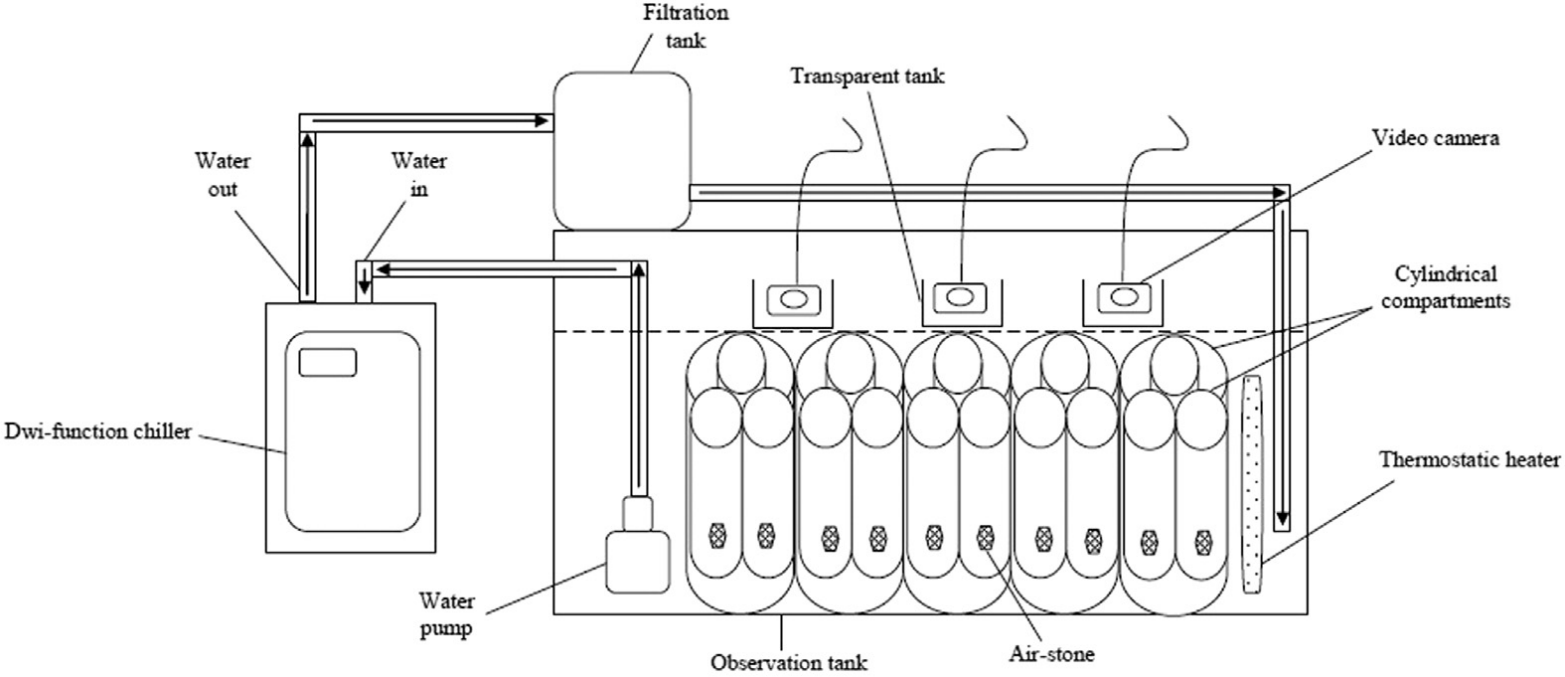
A diagram showing the re-circulating marine aquaculture system equipped with video camera used in the present study.

Five water-circulating systems (500 L) were setup in the hatchery to maintain five different water temperatures. Systems were connected with the dwi-function chiller and thermostatic heater to determine the acclimation temperature (Fig. 2). Chillers were purchased from Hailea Model HC-500A and thermostatic heaters were imported from EHEIM Model Jager 300 W. Each tank was equipped with cylindrical compartments of 20.0 cm diameter and 30.5 cm height to place the instar crabs. Video cameras (HDR-CX500E) were purchased from Sony (Malaysia) and were attached above the tanks with transparent aquarium (1 L) (Fig. 2). Cameras were continuously recording and stopped only during feeding time. The molted crabs’ video was transferred to the main computer and if there was no molting, the video was deleted. The large MPEG-TS Video was then uploaded within the Solomon Coder software [1], a simple and free solution for behavior coding [7], [8]. The installation software was freely available online at https://solomoncoder.com/, https://solomoncoder.com/download.php. In this software, the play and fast button were used to forward the recorded video until the molted crabs’ video was found. A timeframe of 30 min was chosen to standardize the behavioral events by rewinding the video of the crabs especially dislocation before molting.

### 2.3 Data processing

Time sequence option was used with the “drawn on video” marking option (available within the software) to mark the locomotor behavior of the crabs 30 min before they molted. Conventional marking calculations were used to calculate the crab movement for further analyses using data processing software of IBM^®^ SPSS^®^ Statistics Version 22 available at https://www.ibm.com/products/spss-statistics. Data was transformed to normality and homoscedasticity before multiple comparison test by post hoc Tukey׳s honest significance difference (Tukey-HSD) was conducted. Additionally, linear regression was calculated to further describe the relationship between temperature and behavioral activity.
